# Quantitative parameters of dynamic contrast-enhanced magnetic resonance imaging to predict lymphovascular invasion and survival outcome in breast cancer

**DOI:** 10.1186/s40644-022-00499-7

**Published:** 2022-10-22

**Authors:** Tianfu Lai, Xiaofeng Chen, Zhiqi Yang, Ruibin Huang, Yuting Liao, Xiangguang Chen, Zhuozhi Dai

**Affiliations:** 1 Department of Radiology, Meizhou People’s Hospital, 514031 Meizhou, China; 2 Guangdong Provincial Key Laboratory of Precision Medicine and Clinical Translational, Research of Hakka Population, 514031 Meizhou, China; 3 Department of Radiology, First Affiliated Hospital of Shantou University Medical College, 515000 Shantou, China; 4 GE Healthcare, 510623 Guangzhou, China; 5 Department of Radiology, Shantou Central Hospital, 515031 Shantou, Guangdong China

**Keywords:** Neoplasm Invasiveness, Prognostic factors, Breast neoplasms, Dynamic contrast-enhanced magnetic resonance imaging

## Abstract

**Background:**

Lymphovascular invasion (LVI) predicts a poor outcome of breast cancer (BC), but LVI can only be postoperatively diagnosed by histopathology. We aimed to determine whether quantitative parameters of dynamic contrast-enhanced magnetic resonance imaging (DCE-MRI) can preoperatively predict LVI and clinical outcome of BC patients.

**Methods:**

A total of 189 consecutive BC patients who underwent multiparametric MRI scans were retrospectively evaluated. Quantitative (*K*^trans^, *V*_e_, *K*_ep_) and semiquantitative DCE-MRI parameters (W_− in_, W_− out_, TTP), and clinicopathological features were compared between LVI-positive and LVI-negative groups. All variables were calculated by using univariate logistic regression analysis to determine the predictors for LVI. Multivariate logistic regression was used to build a combined-predicted model for LVI-positive status. Receiver operating characteristic (ROC) curves evaluated the diagnostic efficiency of the model and Kaplan-Meier curves showed the relationships with the clinical outcomes. Multivariate analyses with a Cox proportional hazard model were used to analyze the hazard ratio (HR) for recurrence-free survival (RFS) and overall survival (OS).

**Results:**

LVI-positive patients had a higher *K*_*ep*_ value than LVI-negative patients (0.92 ± 0.30 vs. 0.81 ± 0.23, *P* = 0.012). N2 stage [odds ratio (OR) = 3.75, *P* = 0.018], N3 stage (OR = 4.28, *P* = 0.044), and *K*_*ep*_ value (OR = 5.52, *P* = 0.016) were associated with LVI positivity. The combined-predicted LVI model that incorporated the N stage and *K*_ep_ yielded an accuracy of 0.735 and a specificity of 0.801. The median RFS was significantly different between the LVI-positive and LVI-negative groups (31.5 vs. 34.0 months, *P* = 0.010) and between the combined-predicted LVI-positive and LVI-negative groups (31.8 vs. 32.0 months, *P* = 0.007). The median OS was not significantly different between the LVI-positive and LVI-negative groups (41.5 vs. 44.0 months, *P* = 0.270) and between the combined-predicted LVI-positive and LVI-negative groups (42.8 vs. 43.5 months, *P* = 0.970). LVI status (HR = 2.40), N2 (HR = 3.35), and the combined-predicted LVI model (HR = 1.61) were independently associated with disease recurrence.

**Conclusion:**

The quantitative parameter of *K*_*ep*_ could predict LVI. LVI status, N stage, and the combined-predicted LVI model were predictors of a poor RFS but not OS.

**Supplementary Information:**

The online version contains supplementary material available at 10.1186/s40644-022-00499-7.

## Background

Lymphovascular invasion (LVI), defined as the infiltration of tumor cells into lymphatic or blood vessels at the periphery of invasive carcinoma [1–3], has been widely recognized as a negative prognostic factor in multiple cancers, such as breast cancer (BC), gastric cancer, and rectal cancer [4–7]. Even so, LVI has not been included as an important parameter to consider before adjuvant chemotherapy in the National Comprehensive Cancer Network guidelines for BC due to its hard to determine before adjuvant chemotherapy, which can only be postoperatively diagnosed by histopathology. Thus, more evidence is needed to warrant the application of LVI in clinical decision-making.

As a significant preoperative examination for BC patients, dynamic contrast-enhanced magnetic resonance imaging (DCE-MRI) are effective tools for evaluating tumor microcirculation [2, 8–13]. The correlation between tumor microcirculation and LVI have been demonstrated and assessed by qualitative parameters of breast MRI in several small cohort studies [14–17]. However, few studies have been published on the prediction of LVI using large samples of quantitative DCE-MRI parameters. In addition, the relationship between the prediction model of LVI and the prognosis of BC is unclear.

Thus, the purpose of this retrospective study was to determine whether quantitative parameters of DCE-MRI can preoperatively predict LVI and to investigate the relationship between the prediction model and survival in terms of disease-specific recurrence and mortality in BC patients to guide clinical decision-making.

## Materials and methods

### Basic characteristics

This retrospective study was approved by our institutional review, and it waived the informed consent requirement. Between January 2016 and March 2019, 460 consecutive women with BC confirmed by postoperative histopathology had undergone preoperative DCE-MRI of the breast. Their medical charts were reviewed. The patient exclusion criteria were shown as follows: (1) patients with previous neoadjuvant treatment; (2) patients with recurrence of BC; (3) patients without obvious lesions on breast MRI; (4) patients with poor quality MRI images; (5) patients with a maximum tumor diameter < 1.0 cm;6) patients with non-mass-like enhancement lesions; and 7) patients with bilateral lesions or multiple lesions.The patient inclusion and exclusion criteria were shown in **Figure E1** in the supplementary materials. The study patients are part of a large retrospective DCE-MRI database, of which 165 patients have been reported in a previously published study [18]. A previous study only explored the ability of DCE-MRI to predict BC receptor status and molecular subtypes, rather than LVI and survival outcome [18]. Cinicopathological data, inculding age, tumor size (maximum, minimum, and effective diameter), progesterone receptor (PR), estrogen receptor (ER), human epidermal growth factor receptor2 (HER2), Ki-67, molecular subtypes, tumor grade, tumor node metastasis(TNM) stage, and postoperative treatment methods (radiation therapy, adjuvant endocrine therapy, and adjuvant chemotherapy) were used to predict LVI. The clinicopathological data except receptor status and age were no used in the previous study. In addition, follow-up information were added for survival analysis of LVI.

Effective tumor diameter was defined as the mean value of the maximum and minimum tumor diameter. The TNM stage was reclassified based on the 8th edition of the American Joint Committee on Cancer (AJCC) staging manual. LVI was defined as tumor cells present within a definite endothelial-lined space (either lymphatic or blood vessel) that was only visible on microscopy. Additionally, the immunohistochemistry (IHC) technique was used to classify the status of ER, PR, HER2, and Ki-67 [19].The standard of cut off values for the positivity of Ki-67, HER-2, PR, and ER were all based on internationally recognized standards [13].

### Definition of recurrence-free survival and follow-up

According to the follow-up protocol of our hospital, all patients were postoperatively followed up with chest and abdominal CT, and breast mammography every 12 months. Additionally, the patients with mastectomy were postoperatively followed up with breast, abdominal, gynecological, and superficial lymph node ultrasound every 3 months for the first two years. Of the contralateral breast usually followed up with breast mammography and ultrasound. The patients with breast-conserving surgery were postoperatively followed up with breast MRI every 12 months.

Recurrence-free survival (RFS) was calculated in months from the date of surgery to the first date of local recurrence, distant metastasis, February 28, 2021, or death, whichever came first. Overall survival (OS) was calculated in months from the date of surgery to the date of death or on February 28, 2021, whichever came first.

### MRI examination

Breast MRI examinations were performed on a 3.0 T MR scanner (Magnetom Skyra, Siemens Healthcare, Erlangen, Germany) using a 16-channel bilateral breast coil with the patient in the prone position. Before dynamic scanning, T1-weighted volume interpolated breath-hold examination (VIBE) was first performed at two different flip angles 2° and 15° to calculate the T1-mapping images (TR = 3.78 ms, TE = 1.38 ms, FOV = 340 mm×340 mm, matrix = 205 × 256, slice thickness = 2 mm, voxel resolution = 1.3 mm×1.3 mm×2.0 mm, acquisition time = 84 s), and then dynamic scanning with 34 consecutive phases was performed using a combination of VIBE with controlled aliasing in parallel imaging results in higher acceleration (CAIPIRINHA), view-sharing time-resolved imaging with interleaved stochastic trajectories (TWIST), and Dixon fat suppression (CAIPIRINHA-Dixon-TWIST-VIBE) sequence. CAIPIRINHA-Dixon-TWIST-VIBE was used for DCE MRI acquisition, and 2 echoes were used for Dixon-based fat suppression. The DCE-MRI scanning time was 17.7s after injection.The scanning parameters of DCE-MRI included TR = 6.4 ms, TE = 3.34 ms, FOV = 340 mm×340 mm, matrix = 288 × 384, voxel resolution was 0.9 mm×0.9 mm×2.0 mm, slice thickness = 2 mm, slices = 80, no slice gap, flip angle 9°, PAT factor 4, partial Fourier factor, temporal resolution 8.7 s (for one phase), and acquisition time 305 s. An intravenous bolus injection of gadopentetate dimeglumine (Bayer Pharma AG ) with a dose of 0.2 ml/kg was power injected at a rate of 3.0 ml/s, followed by a 20 ml saline injection.

### MR image analysis

The tumor size was measured on the largest section of the tumor on DCE-MRI images. MRI parameters were measured independently by two radiologists (with more than ten years of experience in breast MRI imaging) who were both blinded to the patients’ clinical history and other examination results. All MRI image data were transferred to a workstation, and BC was identified in DCE-MRI images as the prominent area of enhancement area. DCE-derived parametric maps were automatically generated after motion correction, and registration was performed by using Tissue 4D software (Siemens Healthcare). First, three maximum continuous sections of tumor on DCE-MRI images were selected, and then regions of interest (ROIs) with a minimum area of 10 mm^2^ were manually drawn on those sections, avoiding visible necrosis, obvious bleeding, vessels, calcifications, and cystic appearing areas. The parameters, including the volume transfer constant (*K*^trans^, min^− 1^), extracellular-extravascular volume fraction (*V*_e_, min^− 1^), reverse reflux rate constant (*K*_ep_=*K*^trans^/*V*_e_), rate of contrast enhancement for inflow (W-in, min^− 1^), rate of contrast decay for outflow (W-out, min^− 1^), and the time to peak enhancement after injection (TTP, min) were generated for each voxel defined by the ROIs. The pharmacokinetic parameters were analyzed based on Tofts model of Tissue 4D software (Siemens Healthcare) with a population average arterial input function (AIF) (“intermediate” type). The mean values of the parameters were used for further statistical analysis.

### Statistical analysis

The statistical analysis was performed with R (version 3.6.0). A two-tailed *P* < 0.05 was considered to reflect statistical significance. Interobserver agreement for the DCE-MRI parameters between two radiologists was assessed using the intraclass correlation coefficient (ICC). An ICC value of 0.75~0.90 indicates good reliability, while an ICC value ≥ 0.90 indicates excellent reliability [2, 20].

Continuous variables (age, tumor size, *K*^*trans*^, K*ep, V*_e,_ W-in, W-out, TTP) with a normal distribution are reported as the means ± standard deviation and were compared with the Student’s *t*-test, while categorical variables (ER,PR, HER2, Ki-67, tumor grade, molecular subtype, TNM stage, AJCC stage) are summarized as frequencies and percentages and were compared with the chi-squared test (for nominal variables) and the Kruskal-Wallis *H* test (for ordinal variables) between LVI-positive and LVI-negative groups. The odds ratio (OR) with a 95% confidence interval (CI) for all variables was calculated by using univariate logistic regression analysis to determine the predictors for LVI-positive status. In addition, the combined prediction model for LVI-positive status was constructed by using multivariate logistic regression, and receiver operating characteristic (ROC) curve analysis was performed to evaluate the diagnostic efficiency of the model. The area under the curve (AUC), accuracy, sensitivity, and specificity were calculated, and the optimal cutoff point of the variables was determined according to the largest Youden index.

For survival analysis, survival curves of LVI and predicted LVI were generated by using the Kaplan-Meier method and compared by the log-rank test. Multivariate analyses with a Cox proportional hazard model were used to analyze the hazard ratio (HR) with 95% CI for RFS and OS by using the statistically significant variables (*P* < 0.05) from the univariate Cox analysis.

## Results

### Patient characteristics

**Fig. 1:**
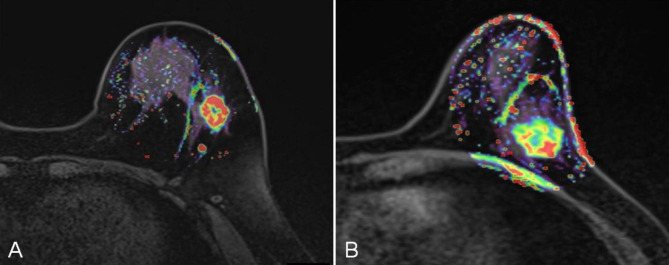
Representative *K*_ep_ images from breast cancer patients with (A) lymphovascular invasion (LVI) and without (B) LVI. Red represents high values of *K*_ep_, yellow represents intermediate values of *K*_ep_, and blue represents low values of *K*_ep_. The *K*_ep_ values of the tumors were 1.05 min^− 1^ and 0.757 min^− 1^, respectively

Finally, 189 women (mean age: 51.1 years; range:25~95 years) were included in this study. Out of 189 included women, 43 had LVI-positive tumors and 146 had LVI-negative tumors. The characteristics of patients in the LVI-negative and LVI-positive groups are shown in **Table E1** in the supplementary materials. Those pertinent features are presented in Table 1. The occurrence rates of lymphatic metastasis was more higher in LVI-positive patients than in LVI-negative patients (*P* = 0.003). In addition, the LVI-positive patients had higher *K*_*ep*_ values than the LVI-negative patients (0.92 ± 0.30 vs. 0.81 ± 0.23, *P* = 0.012, Fig. 1). No significant differences were found for age, tumor size, ER, PR, HER2, Ki-67 index, tumor grade, molecular subtype, T stage, M stage, AJCC stage, *K*^*trans*^, *V*_e,_ W-in, W-out, or TTP value between patients with and without LVI (all *P* > 0.05).

**Table 1:** The characteristics of the patients in the LVI-negative and LVI-positive groups

	LVI- (n = 146)	LVI+ (n = 43)	*P*	OR (95% CI)	*P* ^*d*^
Maximum diameter (cm)	2.89 ± 1.12	3.09 ± 1.18	0.312^b^	1.16 (0.86–1.55)	0.312
Minimum diameter (cm)	1.85 ± 0.65	1.92 ± 0.72	0.528^b^	1.17 (0.70–1.92)	0.526
ER			0.837^a^		
Negative	50 (34.25%)	14 (32.56%)		1	NA
Positive	96 (65.75%)	29 (67.44%)		1.08 (0.53–2.27)	0.837
PR			0.523^a^		
Negative	70 (47.95%)	23 (53.49%)		1	NA
Positive	76 (52.05%)	20 (46.51%)		0.80 (0.40–1.58)	0.523
HER2			0.845^a^		
Negative	103 (70.55%)	31 (72.09%)		1	NA
Positive	43 (29.45%)	12 (27.91%)		0.93 (0.42–1.94)	0.845
Ki-67			0.409^a^		
≤20%	47 (32.19%)	11 (25.58%)		1	NA
>20%	99 (67.81%)	32 (74.42%)		1.38 (0.66–3.08)	0.410
T stage			0.109^c^		
T0	14 (9.59%)	0 (0.00%)		0.0 (0.0-3.48 × 10 ^13^)	0.988
T1	46 (31.51%)	10 (23.26%)		1	NA
T2	84 (57.53%)	32 (74.42%)		1.75 (0.81–4.05)	0.167
T3	1 (0.68%)	0 (0.00%)		0.0 (NA-Inf)	0.997
T4	1 (0.68%)	1 (2.33%)		4.6 (0.17–123.2)	0.295
N stage			**0.003** ^**c**^		
N0	91 (62.33%)	17 (39.53%)		1	NA
N1	40 (27.40%)	15 (34.88%)		2.01 (0.91–4.43)	0.083
N2	10 (6.85%)	7 (16.28%)		3.75 (1.21–11.2)	**0.018**
N3	5 (3.42%)	4 (9.30%)		4.28 (0.98–17.85)	**0.044**
Parameters					
*K*^*trans*^(min^− 1^)	0.21 ± 0.11	0.22 ± 0.12	0.766^b^	1.58 (0.07–30.11)	0.765
*K*_*ep*_(min^− 1^)	0.81 ± 0.23	0.92 ± 0.30	**0.012** ^**b**^	5.52 (1.42–23.3)	**0.016**
*V*_*e*_	0.27 ± 0.13	0.24 ± 0.09	0.102^b^	0.12 (0.01–2.15)	0.163
W-in (min^− 1^)	0.56 ± 0.22	0.61 ± 0.24	0.228^b^	2.50 (0.56–11.17)	0.228
W-out (min^− 1^)	-0.01 ± 0.02	-0.02 ± 0.02	0.088^b^	0.00 (0.0-9.35)	0.090
TTP (min)	0.69 ± 0.21	0.64 ± 0.17	0.165^b^	0.25 (0.03–1.58)	0.167

Note. *P*^a^: chi-squared test, *P*^b^: Student’s *t*-test, *P*^c^: Kruskal-Wallis H test, *P*^d^: univariate analysis. Abbreviations: ER = Estrogen receptor; PR = Progesterone receptor;HER2 = Human epidermal growth factor receptor2; TNBC = Triple negative breast cancer; NA = Not available.

### Interobserver agreement

The ICC values between two readers for *K*^trans^, *K*_ep_, *V*_e_, W-in, W-out, and TTP were 0.994 (95% CI: 0.992–0.995), 0.858 (95%CI:0.815–0.891), 0.984 (95%CI:0.979–0.988), 0.987 (95%CI: 0.983–0.990), 0.979 (95%CI:0.972–0.984), and 0.961 (95%CI:0.949–0.971), respectively, indicating good or excellent reliability. The coefficient of variation on ICC value is 0.057.

### Predictors of LVI and model development

**Fig. 2:**
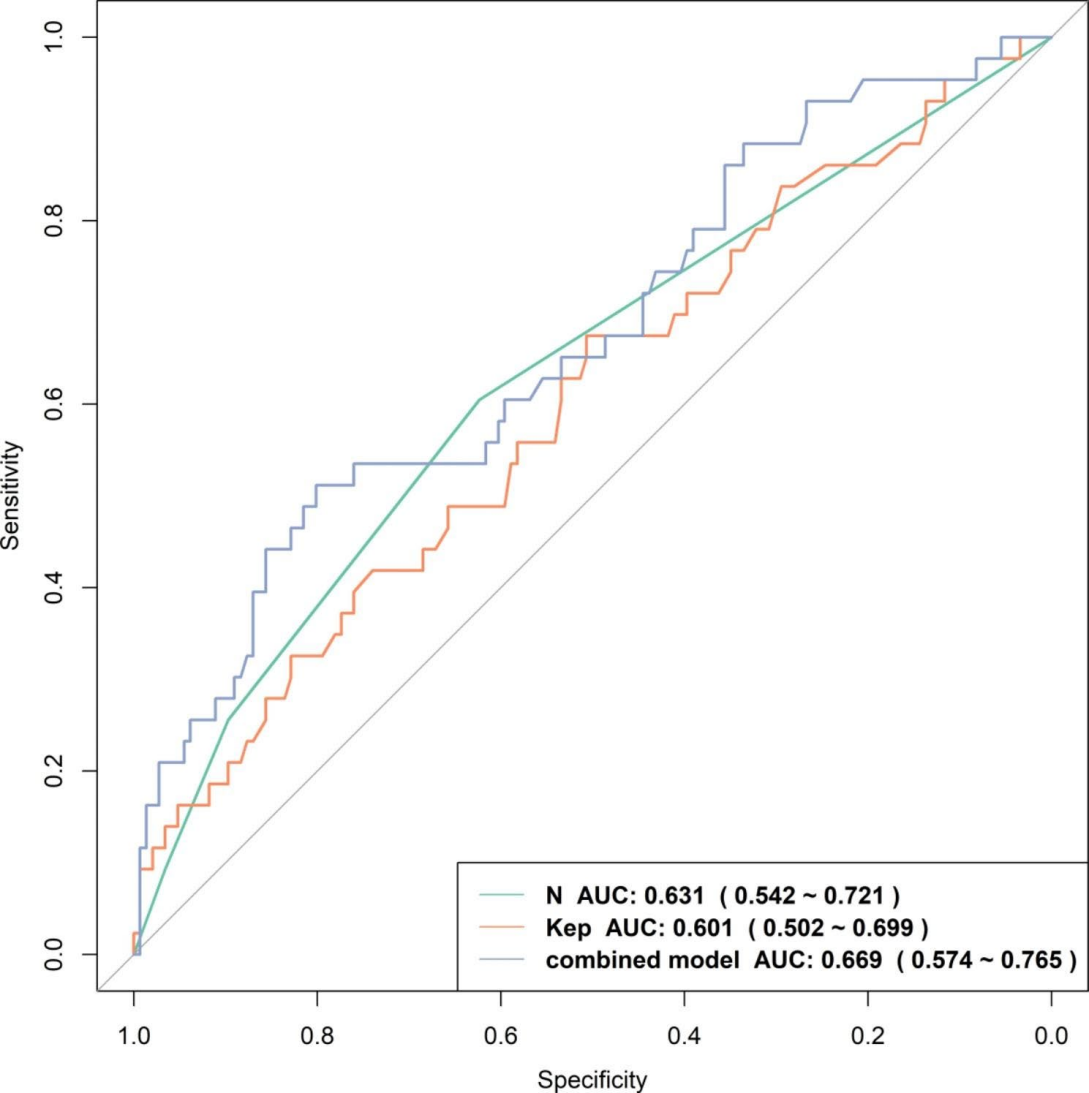
Receiver operating characteristic (ROC) curve of the model established by N stage, *K*_*ep*_, and combined-predicted model that incorporated N stage and *K*_ep_ for the prediction of lymphovascular invasion in breast cancer patients

Univariate logistic regression analysis (**Table E1** in the supplementary materials) showed that N2 (OR = 3.75, *P* = 0.018), N3 (OR = 4.28, *P* = 0.044), and *K*_ep_ value (OR = 5.52, *P* = 0.016) were associated with LVI positivity. ROC curves were generated based on the prediction probability of the regression equation using the above variables (Table 2; Fig. 2). The combined-predicted LVI model that incorporated the N stage and *K*_ep_ yielded a maximum AUC of 0.669 (0.574–0.765), with a cutoff value of 0.276, accuracy of 0.735, sensitivity of 0.512, and specificity of 0.801. Based on the combined-predicted model results, 51 patients were LVI positive and 138 were LVI negative.

**Table 2:** Performance of the individualized prediction models

Models	Cutoff	AUC(95% CI)	Accuracy	Sensitivity	Specificity
N	0.500	0.631 (0.542–0.721)	0.619	0.605	0.623
*K*_ep_	0.808	0.601 (0.502–0.699)	0.545	0.674	0.507
Combined	0.276	0.669 (0.574–0.765)	0.735	0.512	0.801

Note: N and *K*_ep_ indicate the predicted model based on the N stage and *K*_ep_, respectively. Combined indicate the predicted model based on the combination of all above parameters. N mean N2/3 stage versus N0/1 stage.

### Prognostic value of LVI

**Fig. 3:**
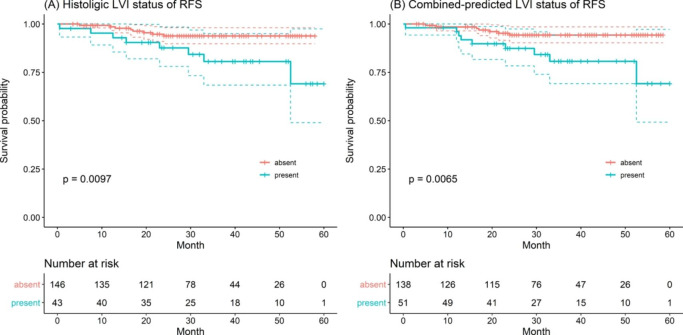
Recurrence-free survival (RFS) curves scaled by histologic lymphovascular invasion (LVI) status (A) and combined-predicted LVI status (B) with Kaplan-Meier analysis

**Fig. 4:**
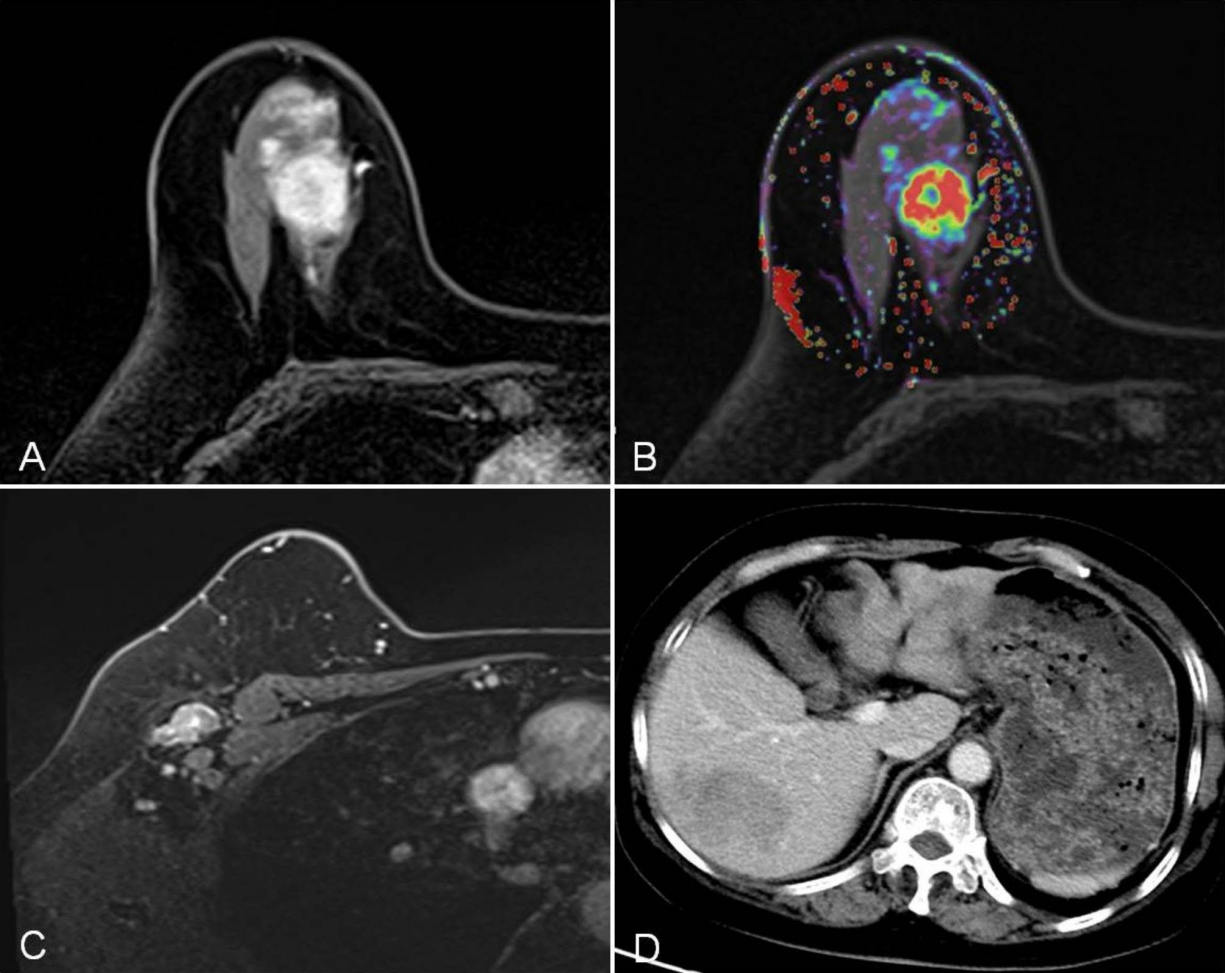
A 59-year-old woman with invasive ductal carcinoma of the right breast. Dynamic contrast-enhanced magnetic resonance imaging (A) showing a solid mass in the upper inner quadrant of the right breast, with nonhomogeneous obvious enhancement, *K*_ep_ value of 1.09 min^− 1^ (B), and lymph node metastasis to the right axilla (C). The LVI risk calculated by the combined model which was built based on the combination of N stage and *K*_ep_ was 70.7%. She was confirmed to be LVI positive by histopathology and had multiple metastatic lesions in the right and left liver lobe on follow-up CT (D) 32.5 months after surgery

As of February 28, 2021, all patients had completed RFS follow-up, and the median RFS of all patients was 32.0 (23.0-42.8) months, of which 16 of 189 (8.47%) had a tumor recurrence. Among 16 patient with recurrences, 4 patients had locoregional recurrence and 12 patients had distant metastases. Of note, the recurrence rate was 18.6% (8/43) in patients with LVI and 5.48% (8/146) in patients without LVI, with a significant difference between the two groups (*P* = 0.016). The median RFS was 31.5 (23.0–42.0) months for patients with LVI and 34.0 (22.0-44.8) months for patients without LVI (log-rank test, *P* = 0.010, Fig. 3 A). Similar results were observed in the combined-predicted LVI model: The median RFS was 31.8 (23.6–58.5) months for patients with combined-predicted LVI presence and 32.0 (21.8–60.0) months for patients with combined-predicted LVI absence (log-rank test, *P* = 0.007, Fig. 3B). According to the results of the univariate Cox regression analysis (**Table E2** in the supplementary materials), LVI (HR = 3.38, *P* = 0.015), N2 (HR = 6.13, *P* = 0.007), and the combined-predicted LVI model (HR = 3.61, *P* = 0.011) were associated with RFS. Multivariate Cox regression analysis showed that N2 (HR = 4.69, *P* = 0.026) was independent predictors of disease recurrence. A representative case is provided to show the discriminative ability of the combined-predicted model for predicting LVI and RFS **(**Fig. 4**)**.

**Fig. 5:**
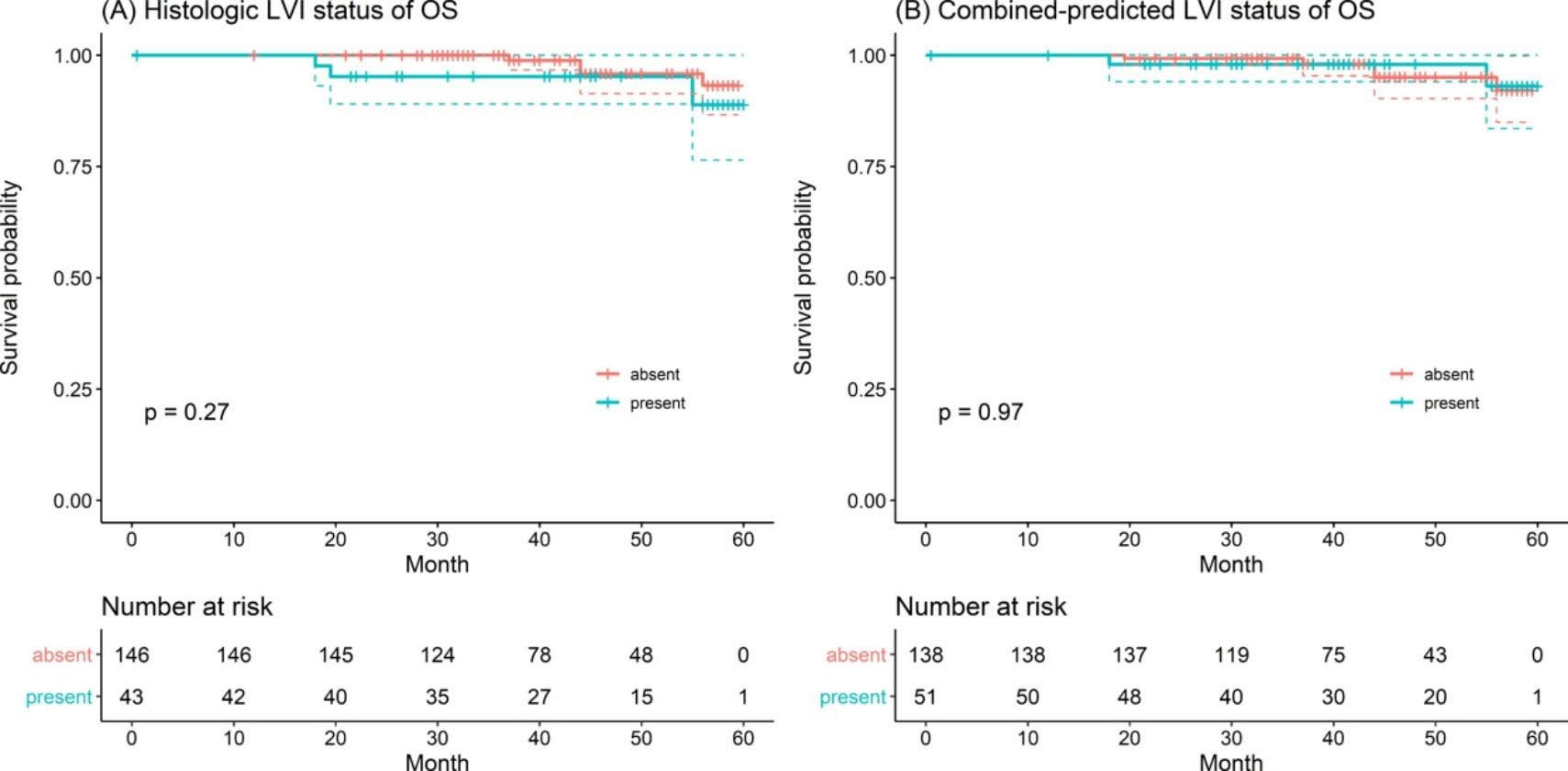
Overall survival (OS) curves scaled by histologic LVI status (A) and combined-predicted LVI status (B) with Kaplan-Meier analysis

When 98.4% (186/189) of patients had completed OS follow-up, the median OS of all patients was 43.0 (31.5–56.5) months, and the overall death rate was 3.70% (7/189). The median OS was 41.5 (32.0-55.5) months for patients with LVI and 44.0 (31.0-57.3) months for patients without LVI (log-rank test, *P* = 0.27, Fig. 5 A), which is consistent with the results of the combined-predicted LVI model: The median OS was 42.8 (32.0-59.5) months for patients with combined-predicted LVI presence and 43.5 (30.8–60.0) months for patients with combined-predicted LVI absence (log-rank test, *P* = 0.970, Fig. 5B). There were no variables significantly associated with OS in the univariate Cox regression analysis (**Table E1** in the supplementary materials, all *P* > 0.05).

## Discussion

In contrast to prior studies, in which simpler qualitative MRI parameters or MRI-based radiomics features were used to evaluate LVI, we developed this novel model based on clinicopathological features and quantitative parameters of DCE-MRI to predict LVI. Our results showed that the N2 stage (OR = 3.75), N3 stage (OR = 4.28), and *K*_*ep*_ value (OR = 5.52) were significantly associated with LVI positivity. The combined-predicted model, which was established by *K*_*ep*_ and N stage, could preoperatively predict LVI status with an accuracy of 0.735 and a specificity of 0.801. The RFS of the LVI-positive group was significantly lower than that of the LVI-negative group (31.5 vs. 34.0 months, *P* = 0.010). Similar results were observed in the combined-predicted LVI-positive group and LVI-negative group (31.8 vs. 32.0 months, *P* = 0.007). The median OS was not significantly different between the LVI-positive and LVI-negative groups (41.5 vs. 44.0 months, *P* = 0.270) and between the combined-predicted LVI-positive and LVI-negative groups (42.8 vs. 43.5 months, *P* = 0.970). LVI, N2, and the combined-predicted LVI model were independently associated with disease recurrence.

The CDT-VIBE sequence is a combination of volumetric T1-weighted CAIPIRINHA-VIBE imaging, TWIST view-sharing, and Dixon fat separation. The great potential of CDT-VIBE for high tempo-spatial resolution breast DCE-MRI has been evaluated and widely acknowledged [21–25]. The parallel imaging (PI) technology (CAIPIRINHA) can further shorten image acquisition time than conventional PI techniques such as sensitivity encoding, and the image signal-to-noise ratio will not be greatly lost [26]. Before calculating the pharmacokinetic parameters, the image was first subjected to motion correction, which can eliminate motion artifacts such as breathing, and then the motion-corrected images were registered to the structural image to ensure that the corresponding tumor levels in different phases were consistent, thereby ensuring ROI consistency. A typical two-compartment Tofts model was used to calculate the pharmacokinetic parameters, and the arterial input function was selected according to the minimum chi-square value to ensure the accuracy and robustness of the pharmacokinetic parameters.

The simpler qualitative parameters, such as internal enhancement pattern, the initial enhancement percentage, maximum enhancement, slope, and kinetic enhancement curve, have been shown significantly correlated with LVI by other investigators, but little attention was paid to the relationship between quantitative parameters of DCE-MRI and LVI. Thus, we developed this novel model to preoperatively predict LVI and clinical outcome of BC patients in this study. *K*_*ep*_, one of the pharmacokinetic parameters of DCE-MRI, is the rate constant of diffusion for contrast agent from the extravascular space into the intravascular space, which depends primarily on the capillary permeability and the permeability surface area [9, 10, 18]. Our study showed that LVI-positive patients had a higher *K*_*ep*_ value than LVI-negative patients (0.92 ± 0.30 vs. 0.81 ± 0.23, *P* = 0.012), and the *K*_*ep*_ value was associated with LVI-positive status (OR = 5.52, *P* = 0.016). This finding is in line with previous findings: LVI strongly correlates with a high peritumoral lymphovascular density and more aggressive neovascularization, and these alterations induce differences in the volume and flow of blood in the tumor microcirculatory environment [2, 6, 9, 27]. However, we found no significant differences in *K*^*trans*^ or *V*_*e*_ between LVI-positive and LVI-negative patients, both of which are important markers of vascular permeability. A possible explanation may be as follows: *K*^*trans*^, K_ep_, and *V*_*e*_ will be affected by multiple factors, including blood perfusions such as cardiac output, hypertension, the circulatory system of an individual, and other physiologic parameters such as tumor cellularity, capillary bed perfusion, microvessel density of the tumors and their permeability [9, 28]. Moreover, perfusion status, such as cardiac output and blood pressure, can easily cause changes in the flow of contrast agents in the tissue, thereby affecting the stability of pharmacokinetic parameters. K^trans^ value measures the joint effect of the plasma flow and tissue permeability properties and is therefore sensitive to these physiological factors. However, K_ep_ tends to be relatively stable in describing actual tumor capillary permeability [28], because it is less sensitive to the absolute value of the contrasts agent concentration. In addition, one thing that probably affect the estimation of *K*^trans^ is the AIF method. Since a population-based AIF is used in this study, it may not be the most accurate one for each individual, and individualized AIF may be more accurate for K^trans[29]^. This may be the reason why we only found a significant difference in K_ep_ but no difference in K^trans^ between LVI-positive and LVI-negative groups. Furthermore, another reason for a correlation with K_ep_ but not with K^trans^ or V_e_ in our study could be due to the complexity of the underlying pathophysiology of heterogeneous BC. Thus, our study showed that *K*_*ep*_ might be a more stable parameter than *K*^*tran*s^ and *V*_e_, consistent with the findings in a previous study [9]. Also consistent with previous studies, our study showed that LVI is a risk factor for axillary and distant metastasis [14, 15, 29, 30].

In the present study, we combined the *K*_*ep*_ value and N stage to predict the histopathology LVI status. ROC analysis showed that the combined-predicted LVI model achieved moderate performance in predicting LVI, with an accuracy of 0.735 and a specificity of 0.801. However, the combined-predicted LVI model overestimates the LVI + case (combined-predicted LVI + 51 vs. histological LVI + 43) based on the cutoff point value determined by the largest Youden index. We used the chi-squared test to analyze these data, and the results showed that there were no significant differences (*P* = 0.405). A similar result was also found in previous studies for a model that predicted LVI in hepatocellular carcinoma (predicted LVI + 176 vs. histological LVI + 115) and gastric cancer patients (predicted LVI + 87 vs. histological LVI + 68) [3, 31]. A potential explanation for this result may be as follow: The cutoff point value determined by the largest Youden index may lead to a higher rate of false-positive for LVI cases. Therefore, more threshold selection strategies and better biomarkers need to be explored to improve the specificity of the prediction model in future research.

In this study, the recurrence rate was significantly higher in patients with LVI than in those without LVI (18.6% vs. 5.48, *P* = 0.016). Multivariate Cox regression analysis showed that LVI (HR = 3.38), N2 (HR = 6.13), and the combined-predicted LVI model (HR = 3.61) were independent predictors of disease recurrence. The median RFS was lower for patients with LVI than for those without LVI (31.5 vs.34.0, *P* = 0.010), which was also observed in the combined-predicted LVI model (31.8 vs.32.0, *P* = 0.007). These results were comparable with previous studies. For instance, Ejlertsen et al. [32] found that disease-free survival was statistically significantly associated with LVI within their high-risk group (HR 2.29, *P* < 0.001). Matsunuma et al. [33] reported that LVI was a significant factor for locoregional recurrence-free survival. However, no significant difference was found for OS between patients with and without LVI or in the combined-predicted LVI model in our study, which was consistent with the results of Rosen et al. [34] and Freedman et al. [35]. Contrary to our findings, Ejlertsen et al. [32] found that OS was statistically significantly associated with LVI. In their study, the cohort had larger and more high-grade tumors, with frequent triple-negative and HER2-positive phenotypes, as compared with our study. These differences might have contributed to the different survival patterns of patients with LVI positive tumors. In addition, we found that N2 but not N3 is an independent predictor associated with RFS, this may be due to fewer patients with N3. In this study, only 9 patients with N3 stage were included in the study, and patients with N3 have a longer median RFS than patients with N2 (32.0 vs. 24.0). Thus, more patients should be included to verify this founding.

The present study had some limitations. First, the study results were assessed in a single institution in a relatively large cohort study with 189 patients, and the inclusion of more patients in a multicenter study will make the results more reliable. Second, LVI status was only classified as positive or negative in this study. Our study did not separate LVI into the vascular invasion and lymphatic invasion. A study by Fujii T et al. [36] found that the presence of vascular invasion but not lymphatic invasion could be an indicator of high biological aggressiveness and maybe a valid prognostic factor for BC. Third, we only used 2D ROIs for the evaluation of BC. It should be noted that 3D ROIs may better reflect the perfusion parameters of the tumors. Fourth, the combined model can successfully predict LVI status with high accuracy and specificity, it have the potential to reduce the need test of combined-predicted LVI negative patients. However, low sensitivity of the combined model needed to be addressed in the future according to the different cut-off point to increase sensitivity in further experiments. Furthermore, there are some limitations in term of survival outcome. First, some patients had only 24 months of follow-up, which may affect the strength of prognostic information. Second, LVI status and the combined-predicted LVI model status were predictors of a poor RFS presumably due to reasonable patient numbers and aggressively treat for recurrence patients. The reasons for these parameters were not predictors of OS presumably due to shorter follow-up time, and we will continue to follow up. In addition, there are some technical limitations in our study. First of all, the classical Tofts model of the dedicated software for pharmacokinetic analysis (TISSUE 4D, Siemens Healthcare) is used for the calculation of PKM parameters. Although the Tofts model is one of the most well-known models and widely used in clinical dynamic enhancement studies [37], it has several defects, such as the ignorance of plasma flow in the region of interest. Secondly, K^trans^ reflects the result of the combined action of capillary permeability and plasma flow in the tissue [38]. Perfusion status is likely to cause changes in the K^trans^ value. Further studies are needed to validate the application of K^trans^ and V_e_ in breast LV I cases. Another technical limitation of our study is the use of a population average arterial input function provided by Tissue 4D. Although we determine the AIF (intermediate type) by the minimum chi-square value of the fitted time concentration curve to ensure the accuracy and robustness of the pharmacokinetic parameters, the population-based AIF is different from the true AIF to some extent, leading to some bias in the results [39].

## Conclusion

In conclusion, the quantitative parameters of *K*_*ep*_ from DCE-MRI, and N2 are independent predictors of LVI in BC patients. LVI status, N stage, and the combined-predicted LVI model were confirmed to predict a poor RFS, but no evidence was found that these features were related to OS.

## Electronic supplementary material

Below is the link to the electronic supplementary material.

Supplementary Material 1

## Data Availability

The data cohorts used and/or analyzed in the present study are available from the corresponding authors upon reasonable request.

## Abbreviations

LVI: Lymphovascular invasion
DCE-MRI: Dynamic contrast enhanced magnetic resonance imaging
BC: Breast cancer
AUC: Area under the curve
OR: Odds ratio
HR: Hazard ratio
RFS: Recurrence-free survival
OS: Overall survival
