# Supplementary material for: Quantitative parameters of dynamic contrast-enhanced magnetic resonance imaging to predict lymphovascular invasion and survival outcome in breast cancer
Source: Cancer Imaging. 2022 Oct 22;22:61. doi: 10.1186/s40644-022-00499-7 (PMC9587620; doi:10.1186/s40644-022-00499-7)
Supplement: Supplementary file 1 — Supplementary Material 1 [file 40644_2022_499_MOESM1_ESM.docx]

**Supplementary Materials**

**Table of Contents:**

1. [Patient selection (Figure E1) 2](#a)

2. [Patient characteristics in LVI-negative and LVI-positive groups (Table E1) 3](#b)

3.[Univariate Cox regression analysis of RFS and OS (Table E2) 5](#c)

# 1. Patient selection

# The patient exclusion criteria were shown as follows: 1) patient with previous neoadjuvant treatment; 2) patients with recurrence of BC; 3) patients without obvious lesions on breast MRI; 4) patients with poor quality MRI image; 5) patients with a maximum tumor diameter <1.0 cm; 6) patients with non-mass-like enhancement lesions; and 7) patients with bilateral lesions or multiple lesions. Figure E1 shows the patient recruitment pathway along with the inclusion and exclusion criteria.


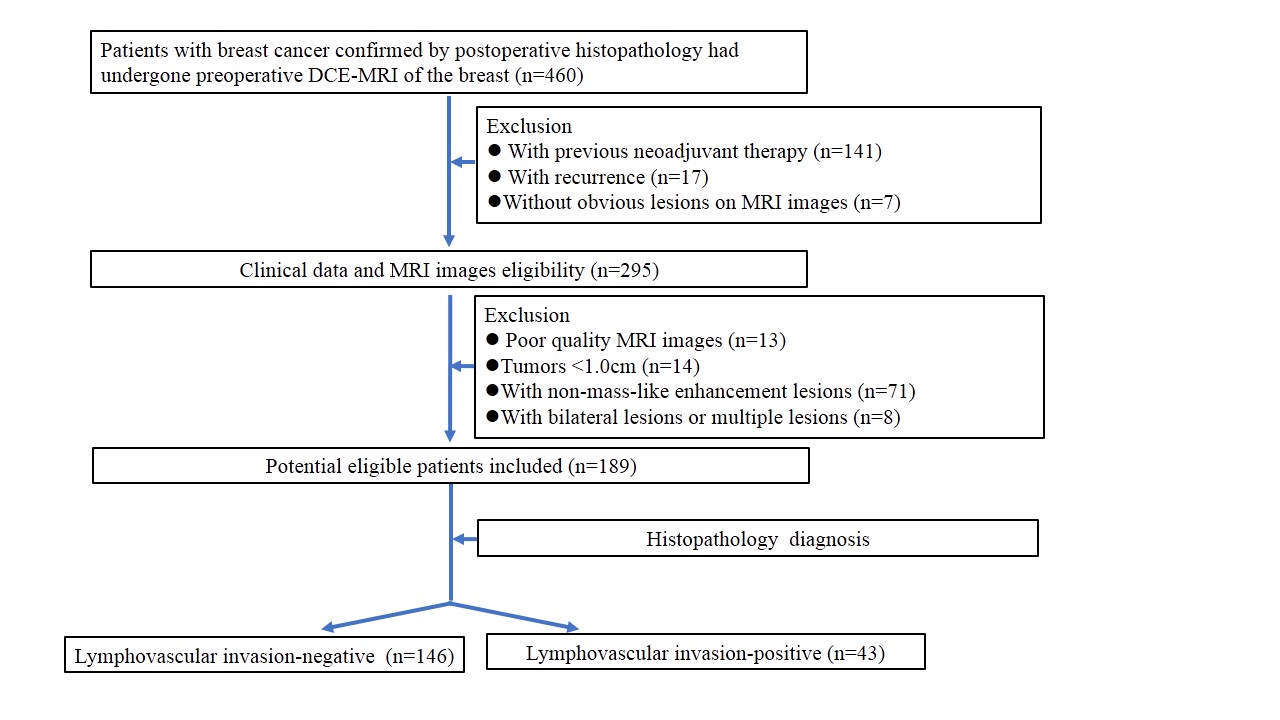


**Figure E1** Patient selection flowchart

# 2. Patient characteristics in LVI-negative and LVI-positive (Table E1)

# Table E1 shows the result of patients characteristics in the LVI-negative and LVI-positive groups

| **Table E1** The characteristics of patients in the LVI-negative and LVI-positive groups | | | | | |
| --- | --- | --- | --- | --- | --- |
|  | LVI- (n=146) | LVI+ (n=43) | *P* | OR (95% CI) | *P^d^* |
| Age (y) |  |  | 0.845^a^ |  |  |
| ≤55 | 103 (70.55%) | 31 (72.09%) |  | 1 | NA |
| ＞55 | 43 (29.45%) | 12 (27.91%) |  | 0.93 (0.42-1.94) | 0.845 |
| Maximum diameter (cm) | 2.89±1.12 | 3.09±1.18 | 0.312^b^ | 1.16 (0.86-1.55) | 0.312 |
| Minimum diameter (cm) | 1.85±0.65 | 1.92±0.72 | 0.528^b^ | 1.17 (0.70-1.92) | 0.526 |
| Effective diameter (cm) | 2.37±0.84 | 2.50±0.87 | 0.354^b^ | 1.20 (0.81-1.77) | 0.353 |
| Effective diameter category (cm) |  |  | 0.489^a^ |  |  |
| ≤3.0 | 119 (81.51%) | 33 (76.74%) |  | 1 | NA |
| ＞3.0 | 27 (18.49%) | 10 (23.26%) |  | 1.34 (0.57-2.97) | 0.490 |
| ER |  |  | 0.837^a^ |  |  |
| Negative | 50 (34.25%) | 14 (32.56%) |  | 1 | NA |
| Positive | 96 (65.75%) | 29 (67.44%) |  | 1.08 (0.53-2.27) | 0.837 |
| PR |  |  | 0.523^a^ |  |  |
| Negative | 70 (47.95%) | 23 (53.49%) |  | 1 | NA |
| Positive | 76 (52.05%) | 20 (46.51%) |  | 0.80 (0.40-1.58) | 0.523 |
| HER2 |  |  | 0.845^a^ |  |  |
| Negative | 103 (70.55%) | 31 (72.09%) |  | 1 | NA |
| Positive | 43 (29.45%) | 12 (27.91%) |  | 0.93 (0.42-1.94) | 0.845 |
| Ki-67 |  |  | 0.409^a^ |  |  |
| ≤20% | 47 (32.19%) | 11 (25.58%) |  | 1 | NA |
| ＞20% | 99 (67.81%) | 32 (74.42%) |  | 1.38 (0.66-3.08) | 0.410 |
| Tumor grade |  |  | 0.740^a^ |  |  |
| II | 119(81.51%) | 36(83.72%) |  | 1 | NA |
| III | 27(18.49%) | 7(16.28%) |  | 0.90(0.45-1.66) | 0.740 |
| Molecular subtype |  |  | 0.683^c^ |  |  |
| Luminal A | 28(19.18%) | 6(13.95%) |  | 1 | NA |
| Luminal B | 68(46.58%) | 23(53.49%) |  | 1.58(0.61-4.64) | 0.371 |
| HER-2 enriched | 19(13.01%) | 7(16.28%) |  | 1.72(0.50-6.12) | 0.390 |
| TNBC | 31(21.23%) | 7(16.28%) |  | 1.05(0.31-3.63) | 0.932 |
| T stage |  |  | 0.109^c^ |  |  |
| T0 | 14 (9.59%) | 0 (0.00%) |  | 0.0 (0.0-3.48×10 ^13^) | 0.988 |
| T1 | 46 (31.51%) | 10 (23.26%) |  | 1 | NA |
| T2 | 84 (57.53%) | 32 (74.42%) |  | 1.75 (0.81-4.05) | 0.167 |
| T3 | 1 (0.68%) | 0 (0.00%) |  | 0.0 (NA-Inf) | 0.997 |
| T4 | 1 (0.68%) | 1 (2.33%) |  | 4.6 (0.17-123.2) | 0.295 |
| N stage |  |  | **0.003^c^** |  |  |
| N0 | 91 (62.33%) | 17 (39.53%) |  | 1 | NA |
| N1 | 40 (27.40%) | 15 (34.88%) |  | 2.01 (0.91-4.43) | 0.083 |
| N2 | 10 (6.85%) | 7 (16.28%) |  | 3.75 (1.21-11.2) | **0.018** |
| N3 | 5 (3.42%) | 4 (9.30%) |  | 4.28 (0.98-17.85) | **0.044** |
| M stage |  |  | 1.000^a^ |  |  |
| M0 | 145 (99.32%) | 43 (100.0%) |  | 1 | NA |
| M1 | 1 (0.68%) | 0 (0.00%) |  | 0.0(NA-2.12×10 ^13^) | 0.988 |
| AJCC stage |  |  | 0.938^c^ |  |  |
| Ⅰ | 28 (19.18%) | 6 (13.95%) |  | 1 | NA |
| Ⅱ | 68 (46.58%) | 23 (53.49%) |  | 1.58 (0.61-4.64) | 0.371 |
| Ⅲ | 19 (13.01%) | 7 (16.28%) |  | 1.72 (0.50-6.12) | 0.390 |
| Ⅳ | 31 (21.23%) | 7 (16.28%) |  | 1.05 (0.31-3.63) | 0.932 |
| Parameters |  |  |  |  |  |
| *K^trans^* (min^-1^) | 0.21±0.11 | 0.22±0.12 | 0.766^b^ | 1.58 (0.07-30.11) | 0.765 |
| *K_ep_*(min^-1^) | 0.81±0.23 | 0.92±0.30 | **0.012^b^** | 5.52 (1.42-23.3) | **0.016** |
| *V_e_* | 0.27±0.13 | 0.24±0.09 | 0.102^b^ | 0.12 (0.01-2.15) | 0.163 |
| W-in (min^-1^) | 0.56±0.22 | 0.61±0.24 | 0.228^b^ | 2.50 (0.56-11.17) | 0.228 |
| W-out (min^-1^) | -0.01±0.02 | -0.02±0.02 | 0.088^b^ | 0.00 (0.0-9.35) | 0.090 |
| TTP (min) | 0.69±0.21 | 0.64±0.17 | 0.165^b^ | 0.25 (0.03-1.58) | 0.167 |
| Note. *P*^a^: chi-squared test, *P*^b^: Student’s *t*-test, *P*^c^: Kruskal-Wallis H test, *P*^d^: univariate analysis. Abbreviations: ER = Estrogen receptor; PR = Progesterone receptor;HER2 = Human epidermal growth factor receptor2; TNBC=Triple negative breast cancer; NA= Not available. | | | | | |

# 3. Univariate Cox regression analysis of recurrence-free survival and overall survival (Table E2)

**Table E2** shows the result of the univariate Cox regression analysis of recurrence-free survival (RFS) and overall survival (OS)

| **Table E2** Univariate Cox regression analysis of recurrence-free survival and overall survival | | | | | |
| --- | --- | --- | --- | --- | --- |
|  | Recurrence-free survival | |  | Overall survival | |
| Variables | Univariate Cox analysis | |  | Univariate Cox analysis | |
|  | HR (95% CI) | *P* |  | HR (95% CI) | *P* |
| Age category |  |  |  |  |  |
| ≤55 | 1 | NA |  | 1 | NA |
| ＞55 | 0.58 (0.17-2.05) | 0.399 |  | 1.70 (0.38~7.59) | 0.490 |
| Maximum diameter | 1.34 (0.94-1.93) | 0.110 |  | 1.26 (0.73-2.15) | 0.410 |
| Minimum diameter | 1.58 (0.82-3.0) | 0.171 |  | 2.04 (0.76-5.50) | 0.158 |
| Effective diameter | 1.52 (0.92-2.51) | 0.105 |  | 1.53 (0.72-3.25) | 0.273 |
| Effective diameter category | |  |  |  |  |
| ≤3.0 | 1 | NA |  | 1 | NA |
| ＞3.0 | 1.81 (0.63~5.24) | 0.273 |  | 2.39 (0.53~10.82) | 0.257 |
| ER |  |  |  |  |  |
| Negative | 1 | NA |  | 1 | NA |
| Positive | 0.99 (0.34-2.86) | 0.998 |  | 0.54 (0.12~2.43) | 0.422 |
| PR |  |  |  |  |  |
| Negative | 1 | NA |  | 1 | NA |
| Positive | 0.745 (0.28-2.00) | 0.559 |  | 0.70 (0.16~3.14) | 0.644 |
| HER2 |  |  |  |  |  |
| Negative | 1 | NA |  | 1 | NA |
| Positive | 1.42 (0.52-3.90) | 0.499 |  | 0.97 (0.19~5.00) | 0.970 |
| Ki-67 |  |  |  |  |  |
| <20% | 1 | NA |  | 1 | NA |
| ≥20% | 1.03(0.36-2.97) | 0.957 |  | 1.32(0.26~6.83) | 0.740 |
| LVI |  |  |  |  |  |
| Negative | 1 | NA |  | 1 | NA |
| Positive | 3.38 (1.27-9.02) | **0.015** |  | 2.28 (0.51-10.19) | 0.282 |
| Radiation therapy |  |  |  |  |  |
| No | 1 | NA |  | 1 | NA |
| Yes | 1.11 (0.39~3.20) | 0.846 |  | 0.54 (0.07~4.52) | 0.570 |
| Adjuvant endocrine therapy | |  |  |  |  |
| No | 1 | NA |  | 1 | NA |
| Yes | 1.53 (0.53~4.41) | 0.433 |  | 1.52 (0.18~12.64) | 0.700 |
| Adjuvant chemotherapy | |  |  |  |  |
| Yes | 1 | NA |  | 1 | NA |
| No | 1.17 (0.33~4.11) | 0.809 |  | 0.84 (0.19~3.79) | 0.825 |
| T stage |  |  |  |  |  |
| T0 | 1 | NA |  | 1 | NA |
| T1 | 31343317(0.00~Inf) | 0.998 |  | 38681563 (0.00~Inf) | 0.999 |
| T2 | 110604032 (0.00~Inf) | 0.998 |  | 100783053 (0.00~Inf) | 0.999 |
| T3 | 1.00 (0.00~Inf) | 1.000 |  | 1.00 (0.00~Inf) | 1.000 |
| T4 | 1.00 (0.00~Inf) | 1.000 |  | 1.00 (0.00~Inf) | 1.000 |
| N stage |  |  |  |  |  |
| N0 | 1 | NA |  | 1 | NA |
| N1 | 1.99 (0.58~6.90) | 0.276 |  | 2.90 (0.48~17.34) | 0.244 |
| N2 | 6.13 (1.64~23.00) | **0.007** |  | 2.72 (0.25~30.16) | 0.414 |
| N3 | 4.31 (0.83~22.41) | 0.083 |  | 4.52 (0.40~50.76) | 0.221 |
| M stage |  |  |  |  |  |
| M0 | 1 | NA |  | 1 | NA |
| M1 | 0.00 (0.00-inf) | 0.998 |  | 0.00 (0.00~Inf) | 0.999 |
| AJCC stage |  |  |  |  |  |
| Ⅰ | 1 | NA |  | 1 | NA |
| Ⅱ | 1.56 (0.34~7.24) | 0.569 |  | 81929952 (0.00~Inf) | 0.998 |
| Ⅲ | 1.34 (0.19~9.52) | 0.770 |  | 87300483 (0.00~Inf) | 0.998 |
| Ⅳ | 1.50 (0.25~8.95) | 0.660 |  | 134826073 (0.00~Inf) | 0.998 |
| *K^trans^* | 1.06 (0.01-88.90) | 0.979 |  | 3.05 (0.01-1155.13) | 0.713 |
| *K_ep_* | 2.68 (0.52-13.83) | 0.239 |  | 0.76 (0.04-13.16) | 0.852 |
| *V_e_* | 0.22 (0.003-17.08) | 0.497 |  | 13.86 (0.03-6470.88) | 0.402 |
| W-in | 4.44 (0.58-33.89) | 0.150 |  | 4.76 (0.27-85.34) | 0.290 |
| W-out | 0.00 (0.00-5.39) | 0.067 |  | 0.00 (0.00-13622446779) | 0.497 |
| TTP | 0.331 (0.02-5.76) | 0.448 |  | 0.97 (0.02-39.08) | 0.989 |
| Combined-predicted LVI model | 3.61 (1.34-9.69) | **0.011** |  | 1.14 (0.22-5.92) | 0.873 |
| Abbreviations: ER = Estrogen receptor; PR = Progesterone receptor;HER-2 = Human epidermal growth factor receptor-2; LVI = Lymphovascular invasion; NA= Not available; HR = Hazard ratio; CI = Confidence interval. | | | | | |
